# From the Idea to Its Realization: The Evolution of Minimally Invasive Techniques in Neurosurgery

**DOI:** 10.1155/2013/171369

**Published:** 2013-12-17

**Authors:** P. Grunert

**Affiliations:** Neurosurgical Department, University of Saarland, 66424 Homburg, Germany

## Abstract

Minimally invasive techniques in neurosurgery evolved in two steps. Many minimally invasive concepts like neuronavigation, endoscopy, or frame based stereotaxy were developed by the pioneers of neurosurgery, but it took decades till further technical developments made the realization and broad clinical application of these early ideas safe and possible. This thesis will be demonstrated by giving examples of the evolution of four minimally invasive techiques: neuronavigation, transsphenoidal pituitary surgery, neuroendoscopy and stereotaxy. The reasons for their early failure and also the crucial steps for the rediscovery of these minimally invasive techniques will be analysed. In the 80th of the 20th century endoscopy became increasingly applied in different surgical fields. The abdominal surgeons coined as first for their endoscopic procedures the term minimally invasive surgery in contrast to open surgery. In neurrosurgery the term minimally invasive surgery stood not in opposiotion to open procedures but was understood as a general concept and philosophy using the modern technology such as neuronavigation, endoscopy and planing computer workstations with the aim to make the procedures less traumatic.

## 1. Introduction

**Despite success in surgical technique permitting to perform hemicraniotomy for removing a small intracranial lesion, one cannot but admits, both a priori and from clinical experience, the necessity to minimize surgical injury and approve all methods of precise localization of a cerebral lesion. GI Rossolimo, J Neuropath Psych Korsakow 1907; 7: p 640.**

This statement of Rossolimo, one of the leading Russian neurologists at the beginning of the 20th century, shows that the idea of minimally invasive neurosurgery was already appreciated by the pioneers of this discipline.

Atraumatic operative techniques were considered from the beginning in neurosurgery due to the disastrous possible functional complications inherent to manipulations on the central nervous system. However due to technical limitations most of the promising projects and concepts had to be postponed at the end of the 19th and beginning of 20th century and had to wait sometimes even many decades till technical developments, necessary diagnostic radiological imaging, and the appropriate operative instruments made a safe clinical application of these nearly forgotten ideas in the second half of the 20th century possible. An example for an extreme long time-lag between an idea and its technical realization may serve the development of automata and robots. The main concept of these devices was already described in the Iliad of Homer 2700 years ago. Thetis, the mother of Achilles, arrived in the smithy of the God Hephaistos and asked for the new armour for her son. She was filled with wonder about two types of marvellous machines there. The one called automata were vehicles on three wheels driven by themselves and bringing food and drinks intelligently to the Olympic deities [1]. The others were beautiful female robots in form of androids from gold which were able to speak and sing and which supported God Hephaistos in his smithy [2]. After 2700 years, the present advanced computer technology and progress in artificial intelligence realized this ancient human dream and concept.

In this paper, I will demonstrate my thesis of a two-step development of minimally invasive neurosurgical techniques giving examples from four neurosurgical fields:

1. localization of intracerebral lesions and eloquent cortical areas,
2. transsphenoidal approach to the pituitary tumours,
3. endoscopic treatment of hydrocephalus,
4. functional stereotaxic neurosurgery.

## 2. Localization of Intracerebral Lesions and Eloquent Cortical Areas

The existence and the localization of specific cognitive functions of cortical areas such as movement, speech, or vision were of great importance for the pioneers of neurosurgery to omit severe postoperative side effects such as hemiparesis or aphasia in the mid 19th century. At this time not only the precise localization of brain functions in particular the higher cognitive functions but even their morphological existence was an open question. Some experimental studies spoke in favour of the localization theory; others such as the results of the biologist Jean Pierre Flourens (1794–1867) performed on rabbits and pigeons were compatible with a global representation of cognitive functions distributed over the whole cortex [3].

**Figure 1:**
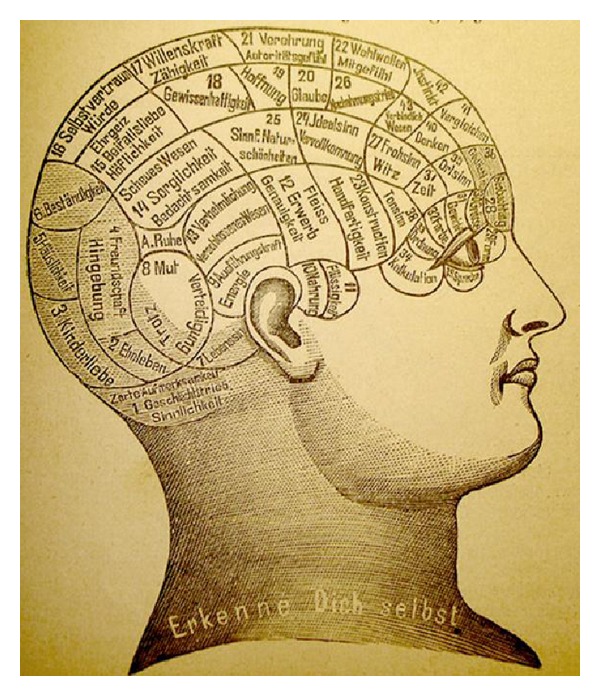
Gall's phrenology: the cortex is subdivided in several independent areas with particular functions of cognition and behavior.

A precursor of the localization of eloquent cortical areas constituted the phrenology developed by the Viennese physician Franz Joseph Gall (1758–1828) at beginning of 19th century [4]. Gall subdivided the cortex initially in 27 and later his pupil Johannes Spurzheim (1776–1832) in 37 separated independently functioning areas which were responsible for different faculties (Figure 1). Besides cognitive functions such as intelligence, memory, or the ability to recognize the size of the objects, Gall associated in a very speculative way based on anatomy of the skull also personality features such as parental love, belief in religion, idealism, or benevolence with distinct cortical areas. He was convinced that pronounced features or the character of a person had a strict morphologic correlate leading to a hypertrophy of the corresponding cortical area and the skull beyond [4]. He believed also that in reverse by palpation of the external bony bumbs on the hypertrophy of specific functional cortical areas and thus on the personality and character of the person can be concluded. Phrenology spread with Spurzheim to UK and to the United States and became an amusing subject in the salons of the upper class society in the first half of the 19th century. Despite the fact that from our present scientific point of view this theory is obsolete, it has to be admitted in favour of Gall that he was one of the first physicians who again underlined the significance of the brain and who initiated further study on the localization of the higher cognitive brain functions. However at the academic level, the location of brain functions in particular the higher cognitive abilities remained an unsolved issue in that time. Over time an increasing opposition against phrenology arouse from scientists in particular by Flourens [3] who was asked by the French academy of sciences to investigate scientifically the propositions of Gall's theory. Challenged by Gall's assumptions and due to increasing withdrawing from a romantic natural philosophy toward measurable objective science of nature, an intense study of cortical functions with anatomical, histological, and electrophysiological methods started to develop in the mid 19th century. It is not surprising therefore that also pioneers of neurosurgery among others such as Victor Horsley (1857–1916) participated in this research themselves and investigated experimentally the localization of cognitive functions.

The first breakthrough in favour of the localization theory was the observations of the French anatomist, surgeon, and anthropologist Paul Broca (1824–1880) who was able to demonstrate that patients with a severe speech disturbance have a lesion in their frontoopercular cortical area in the left dominant hemisphere. First, he described this finding, 1861 after dissection of the brain of a patient known as Tan who died in the hospital where Broca was working as an appointed surgeon. During his life time this patient suffered from a severe speech disturbance and was able only to say the word Tan [5]. In the following years Broca confirmed his initial result on additional 12 patients [6]. His findings were supported in London by the neurologist John Hughlings Jackson (1835–1911) who published a similar case as Broca (1864) [7]. Carl Wernicke (1848–1905), a neurologist and psychiatrist in Breslau, described in his influential work in 1874 “*The aphasic symptoms: a psychological study based on anatomy*” the critical area for understanding the language in the upper temporal gyrus: the sensory speech area [8]. More detailed histological studies of the cortical areas followed by Camillo Golgi (1843–1926) who developed the first staining of neurons [9] and their arborisation. This silver impregnation method enabled Santiago Ramon y Cajal (1852–1934) to investigate in detail the pattern of axonal and dendrite connections of the neuronal tissue [10]. For this work, both scientists were honoured with the Nobel Prize in 1906. The married couple Oscar and Cecilie Vogt (1870–1959 and 1875–1962, resp.) established the first institute dedicated entirely to neuroscience in Berlin where they integrated cytoarchitectonical and electrophysiological techniques for studies of the brain cortex [11]. Korbinian Brodmann (1868–1918) worked at that institute and classified there in the first decade of 20th century the whole cortex into 45 distinct areas based on morphologic characteristics of the grey matter [12]. Besides the pathoanatomical studies, experiments with electrical stimulation became increasingly important for the understanding of cortical function. The first experimental electrical stimulation of the cortex of dogs was performed in 1870 by the two German neuroscientists Julius Eduard Hitzig (1838–1907) and Gustav Theodor Fritsch (1838–1927) [13]. They observed by stimulation of the frontal cortical areas involuntary movements in the contralateral extremities. The experimental Scotch neurologist David Ferrier (1843–1928) published a detailed map of motor functions obtained by stimulation of brain cortex in different animal species in 1876. He published his results under the title “*The function of the brain*” [14]. The significance of these results for neurosurgery was picked by one of the pioneers of neurosurgery in London, Victor Horsley (1857–1916), who already published back in 1887 a map of motor cortical representation based on his experimental study on animals and partly intraoperative studies on humans [15]. Neurosurgery was a unique opportunity to study cortical function intraoperatively also in humans and to obtain important data almost as side effect during the intervention. At the beginning of 20th century the neurophysiologist and later Nobel laureate Charles Scott Sherrington (1857–1952) performed experiments to delineate the motor and the sensory cortex [16]. His map in opposition to previous studies was only a narrow strip on both sides of the Rolandic sulcus [17]. In 1900, Sherrington while working in Liverpool was attended during his experiments for 3 weeks by the promising young American neurosurgeon Harvey Cushing (1869–1939) who was on his educational journey leading him through many important European medical centres. Back home in Baltimore, Cushing applied the cortical stimulation technique on humans during neurosurgical interventions and published a map of sensory cortex in 1908 [18, 19]. Cortical stimulation had also practical implications in neurosurgery and became increasingly important during epilepsy surgery. Fedor Krause (1857–1937), the pioneer of the German neurosurgery, and the neurologist and neurosurgeon Otfrid Foerster (1873–1941) from Breslau, Germany, used the cortical electric stimulation to localize intraoperatively the epileptic foci by provoking an aura or a typical epileptic fit [20–22]. This electrical stimulation was superior to focus localization based merely on anatomical landmarks.

**Figure 2:**
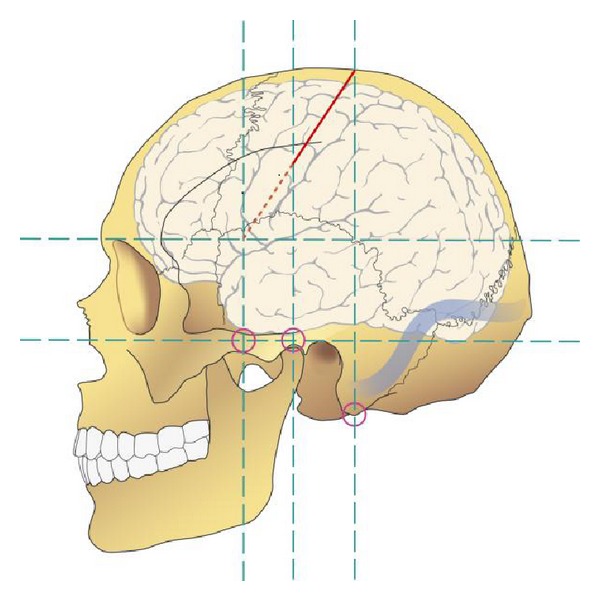
Krönlein's construction of the central region and the Sylvian fissure based on external anatomical landmarks. The horizontal inferior orbitomeatal line is the base line. The parallel supraorbital line marks the inferior border of the central region and the Sylvian fissure. By means of additional three perpendicular vertical lines, first at the anterior zygomatic bone, second at the level through the temporomandibular joint, and the last immediately behind the mastoid, the position of the central region can be drawn into X-ray images or on the head of the patient.

The knowledge of the localization of the eloquent cortical areas had also a very practical consequence for neurosurgery. At the end of the 19th century, two Swiss professors of surgery Rudolf Ulrich Krönlein (1847–1910) [23, 24] in Zurich and Theodor Kocher (1841–1917) [25, 26] in Bern developed independently a method to localize the underneath situated central sulcus and the Sylvian fissure on the scalp. Krönlein used for this a construction of two parallel horizontal and three vertical supporting lines which were based on external bony landmarks (Figure 2) [23]. These lines allowed defining and localizing the position and extension of the central sulcus on the scalp or on the sagittal X-ray image. These supporting lines and the lines representing the central sulcus and the Sylvian fissure in form of ribbons could be also pulled over the head. They marked on the scalp the position of these intracranial structures [24]. The device was called craniometer. In contrast to Krönlein, Kocher's craniometer was based on cadaver studies and consisted of elastic ribbons which were arranged and fixed on the head in a way that the ribbons were just beyond the central sulcus. The elasticity of this craniometer had the advantage that the ribbons preserved their relative position independently of the size of the head. Kocher already described this method in 1892 in his book Lessons in Operative Surgery [25, 26].

**Figure 3:**
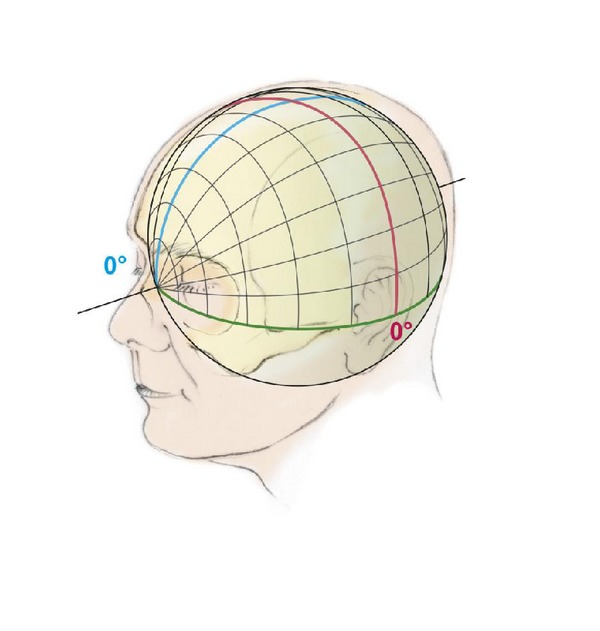
By analogy with a terrestrial globe the superficial coordinates on the head are defined by polar coordinate system by degrees of longitude and latitude. The poles correspond to the nose anterior and to the protuberantia occipitalis posterior, respectively. The (blue) zero meridian is running in the midline from anterior to posterior, dividing the head into a right and a left hemisphere, and the (red) equator divides the head into an anterior and posterior half and is running near the central region.

A more general approach to the problem of localization of cortical structures was developed by the anatomist Dimitri Zernov (1843–1917) [27] and his pupil Nikolay Altukhov [28] at the end of the 19th century in Russia. Zernov called his device an encephalometer. It was a head ring, which was fixed to the patients skull [29, 30]. The basic idea was to understand the head approximately as a terrestrial globe (Figure 3). Every point at the surface of the head was defined similar to the globe by polar coordinates expressed in degrees of latitude and longitude. The position of the globe was in a way that the poles of the globe corresponded to the nose and the protuberantia occipitalis, the zero meridian to the mid sagittal plane along the falx, and the equator to the frontal plane perpendicular to the mid sagittal plane. Thus, the equator was around the central region and divided the head in the frontal plane into two halves. A 2-dimensional map of the brain surface with gyri and sulci with appropriate graticule of meridians and parallels corresponding to a geographical atlas was drawn. The localizations of the cortical structures were established as a mean from measurements of several “normal” cadaver brains. To localize the deep brain structures accurately, a third variable coordinate denoting the distance from the arc centre to the target would be necessary. Since this was not possible with the encephalometer, this apparatus was suitable only for superficially situated lesions. To localize the desired target at the head the frame was equipped with additional two arcs which allowed gliding a stamp like pointer along the meridians and the parallels to set the desired geodetic coordinates on the scalp.

Altukhov published two patients whose central sulcus was successfully localized by this method, and an abscess in this region was subsequently removed in 1891. In a third case the system was used in an inverse modus. The pointer of the encephalometer was set over a visible displaced frontal skull fraction and the geodetic coordinates were read and transferred into the 2D cortical map to identify the affected gyrus [28–30]. Furthermore, the encephalometer was not developed for neurosurgical purposes only. Altukhov used it also for comparative studies to demonstrate possible differences of brains regarding sex, age, and race [28].

Grigorii Ivanovich Rossolimo (1860–1928) [31] from Odessa and Professor of neurology in Moscow, who was one of the founders of paediatric neurology and psychiatry, developed a more sophisticated instrument in 1907 [32]. It was based on the same mathematical principle as the encephalometer, but, instead of a head ring with arcs, it consisted of a helmet in form of a sphere which was fixed to the patient's head and resembled in its mechanical construction the later developed helmet of gamma knife for stereotactic external irradiation. The helmet had in constant distances small holes, arranged along meridians and parallels. It allowed marking with a pointer any target on the scalp without being restricted by the limited movement of the arcs. Rossolimo called this instrument a brain topographer.

However at this stage of neurosurgical development, a broader applicability of these devices was restricted due to insufficient diagnostic imaging capabilities. At that time only a plane X-ray of the head in anterior-posterior and lateral projection was available. Ventriculography was introduced by Walter Dandy (1886–1946) in 1918 [33] and the angiography by the Portuguese neurosurgeon and Nobel Prize laureate Egas Moniz (1874–1955) in 1927 [34]. Therefore, before availability of these two imaging techniques the localization of the lesions was determined entirely by neurologic symptoms of the patient. This circumstance made it necessary to perform the craniotomy large enough to find the lesion at the cortical surface by direct vision or in the subcortical region by palpation with a digit along the brain surface feeling different brain consistence over the lesion. Probably these practical limitations restricted at that time a minimally invasive approach to the lesions and made the application of a brain topographer or encephalometer with few exceptions not practicable. Therefore this original and ingenious method never achieved a general acceptance and fell for many decades into oblivion.

In the 70th and 80th, the diagnostic neuroradiological tools such as CT and MRI were so far advanced that also small intracerebral lesions could be detected inside the intracranial space. The additional fast development of computer technology raised the question whether it is possible to use this computer technology also for real time localization of lesions during neurosurgical interventions without the accurate but time consuming classical frame based stereotaxy. The stereotactic frame restricted additionally the surgical operating field and the mechanical construction of the stereotactic system allowed only limited approaches. The idea to outline in the operating theatre a small craniotomy just above the lesion and to localize the tumour precisely during the intervention without imposing restrictions to the neurosurgeon was finally realized at the end of 80th with the development of neuronavigation devices. These instruments were equipped with a pointer, whose tip could be precisely localized in the space and mapped simultaneously into the corresponding CT/MRI images in real time. The basic mathematics and technology were at this time already extant and used in form of robots [35–38] and GPS navigation and had only to be adapted for the surgical requirements. However robots were already used in neurosurgery some years before neuronavigation systems were applied to neurosurgical localization [39–41].

Robots and global positioning system (GPS) were originally developed by the US army. The robots were developed after the World War II with the aim of working up at distance without human contact radioactively contaminated material, and the GPS was introduced originally for the US navy to navigate ballistic missiles in 1973.

**Figure 4:**
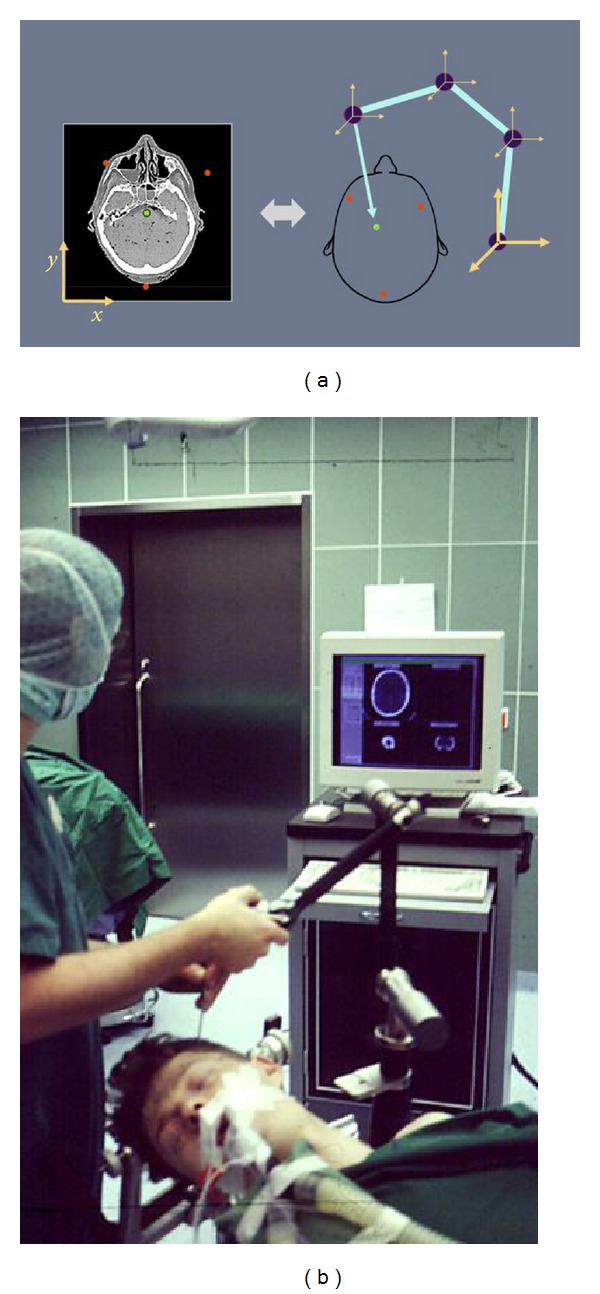
(a) Principle of an arm-based navigation system with calculation of the position from the length of each arm and the angulation of the joints. (b) Arm-based navigation system during intraoperative calibration.

The robotic technology inspired the development of arm-based navigation systems. They calculate their own position in space in a relative homogeneous coordinates system independently of a fixed external point (Figure 4). This is done in real time by evaluation of the angulation in each joint measured by means of encoders integrated in the joint and the a priori known length of each arm between two adjacent joints [42–44].

**Figure 5:**
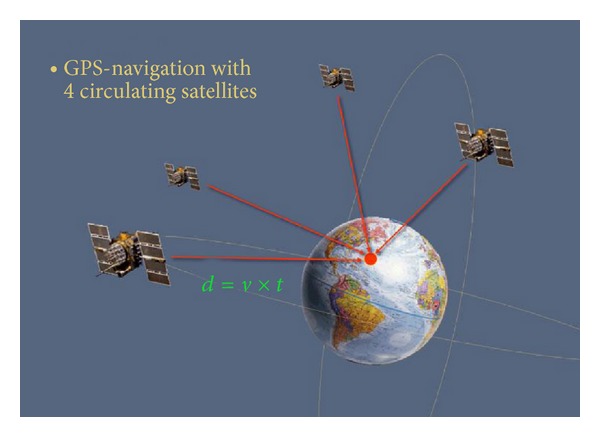
GPS navigation with satellites circulating in an orbit around the earth. From the distances of at least 4 satellites to the receiver, the localization of the receiver can be calculated as the crossing point of spheres with the radius being the distance to the receiver from each satellite.

The armless navigation systems on the other hand were inspired by the GPS. The GPS enables by circulating satellites around the globe the localization of every position at the earth. The principle of the localization consists in measurement of the distances from several satellites to the receiver. Every distance can be understood as a diameter of a sphere. The intersection of these spheres defines the position of the receiver (Figure 5). Each distance is calculated from the known velocity of the electromagnetic wave and the time to reach the receiver. The GPS replaced local navigation systems operating since the World War II by receiving radio signals from fixed navigation beacons. First, satellites were sent into the orbit in 1978, and the full operational capability for the GPS with 24 satellites was reached in 1995. The accuracy of GPS under optimal conditions is about 8 m and is much better than the accuracy of local navigation systems with 180 m. To be not misused by enemies, the accuracy of GPS was till the year 2000 for the public use artificially deteriorated to about 100 m. Thereafter, the undisturbed signal and accuracy were available also for the civil purposes. The GPS was implemented in cars, boots aircrafts, and recently even in cell phones.

**Figure 6:**
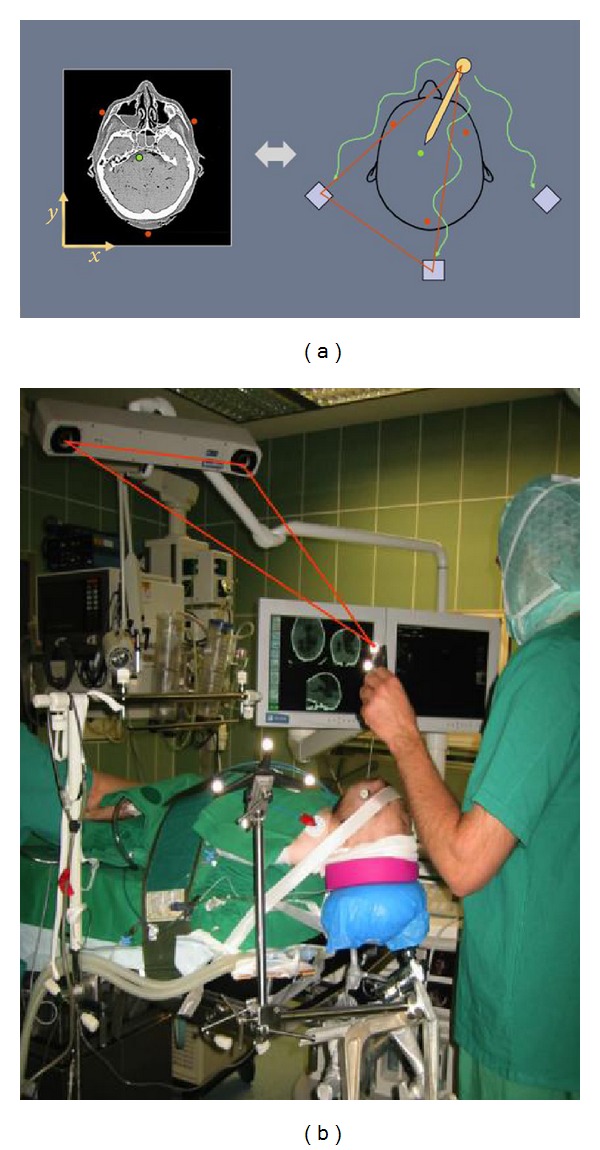
(a) Principle of armless navigation system with emitting infrared diodes on the pointer and a three-camera system as receiver. (b) Armless navigation system with a two-infrared camera system and emitting diodes on the pointer during intraoperative registration. The localization of the pointer is calculated by triangulation method.

The present advanced armless pointer-based neuronavigation systems can be understood as a miniature GPS [45–47]. Like the GPS system, the neuronavigation device calculates distances from several emitting sources whose intersection defines the position in space. The other possibility is to calculate the distance by triangulation technique using at least two fixed cameras (Figure 6). The technical accuracy of the navigation devices to calculate its own position in space is less than 0.5 mm. The emitting sources use either ultrasound infrared light or electromagnetic waves. In GPS system, the position is mapped into a 2D atlas and similarly the calculated position of the pointer tip in the neuronavigation system is transferred into CT/MR image to show in real time the position of the pointer tip during surgery in relation to the anatomical structures visible in the images. To map the pointer tip into the image space, a registration has to be performed first before application. This is done either by paired point registration with touching several corresponding points on the patient and in the image or by matching the skin surface of the image with that of the patient. The serial production and clinical application of neuronavigation devices started in the 90th [45–47]. The operating microscope was also integrated and adapted to be used as an navigation device [48, 49]. Due to the simple handling, the armless pointer based navigation systems gained a general acceptance. They are in the present neurosurgery an indispensable tools for localization of intracranial lesions and minimally invasive craniotomies, exactly as Rossolimo demanded 100 years ago.

## 3. Transsphenoidal Approach to the Hypophysis 

Transsphenoidal pituitary surgery is a further example of a minimally invasive operative technique which was introduced already at the very beginning of the treatment of this pathology, but which became a generally accepted method only in the last 30 years.

At the end of the 19th century endocrinologic disorders started to be related also to the pituitary gland. The French neurologist Pierre Marie (1853–1940) follower of Jean-Martin Charcot in the Hopital Salpetrier in Paris described two patients with acromegaly due to a pituitary tumour in 1886 [50]. Joseph Babinski (1857–1932) [51], a Polish stemming French neurologist in Paris, and the Viennese neurologist and pharmacologist Alfred Fröhlich (1871–1953) [52] published independently of each other two separate cases of an endocrinologically inactive pituitary tumour associated with adipositas and underdeveloped sexual organs later known as dystrophia adiposogenitalis in 1900 and in 1901, respectively. In 1939, Fröhlich fled from the Nazi regime in Austria to the USA and continued his research on the neurovegetative system in Cincinnati, Ohio.

**Figure 7:**
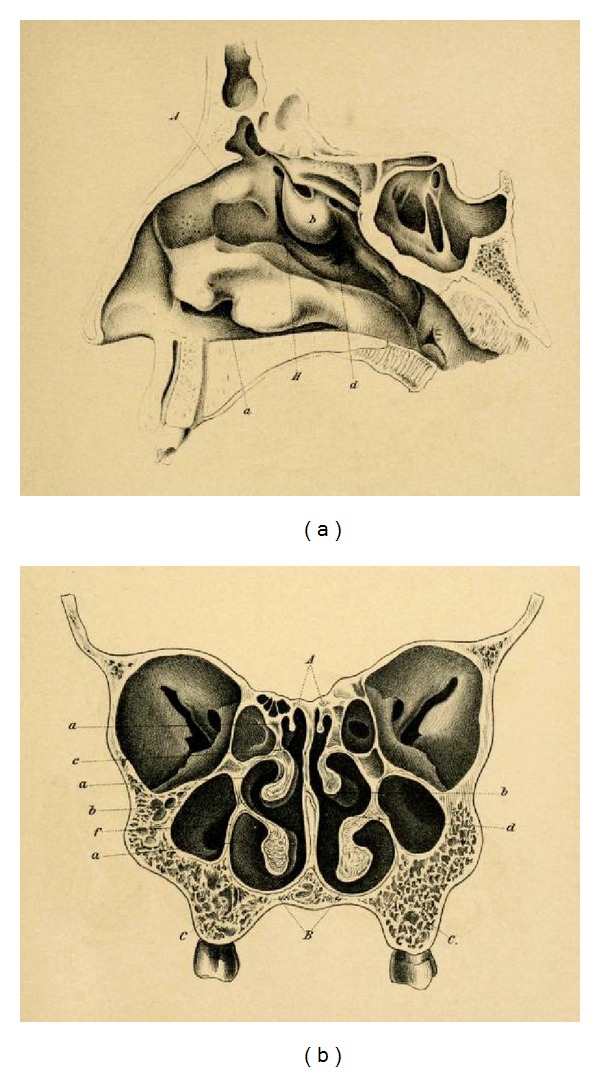
Paranasal sinuses from the publication of Emil Zuckerkandl in 1882: (a) sagittal section through the nose, (b) frontal section through the nose and the maxillary sinuses.

First operations of pituitary tumours were performed by Victor Horsley through a frontal approach in London in 1889, and later in 1904, he described a temporal transcranial approach [53–55]. Fedor Krause (1857–1937) proposed a subfrontal approach in 1905 [55]. His approach was further improved in the United States by Frazier, Heuer, Cushing and Dandy and became the standard transcranial approach in the following years. As an alternative to the transcranial route the transsphenoidal approach was developed simultaneously in the first decade of the 20th century in the United States and in Europe, in particular in the Austrian monarchy. One reason that Vienna became the cradle for minimally invasive approach to pituitary tumours using an endonasal transsphenoidal approach was among others due to the basic and detailed anatomical studies of the paranasal sinuses performed in Vienna by the Austrian anatomist and Violin virtuoso Emil Zuckerkandl (1849–1910). His main work “*On normal and pathological anatomy of the paranasal sinus and its pneumatic adnexes*” in 1882 was the anatomical presupposition for the Viennese ENT surgeons to successfully develop minimally invasive endonasal approaches to pituitary tumours [56] (Figure 7).

**Figure 8:**
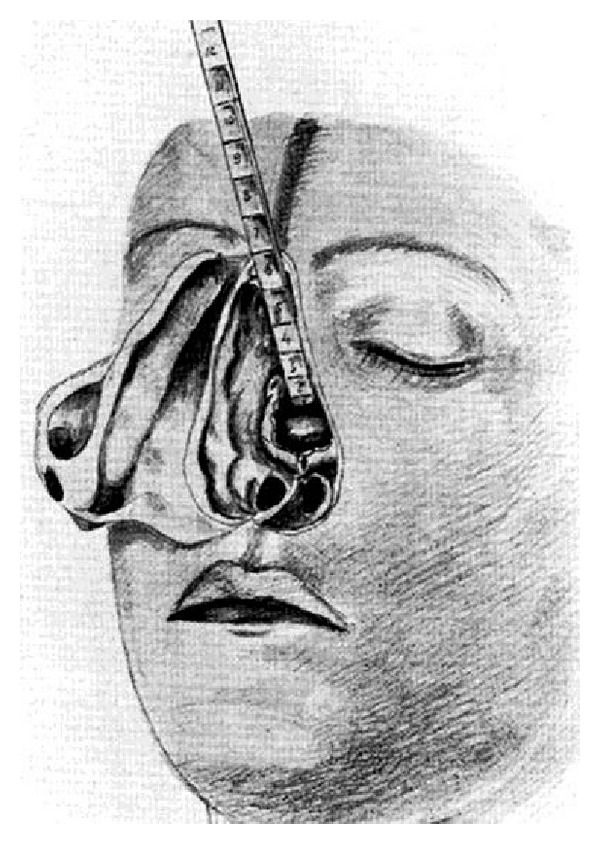
Superior transsphenoidal approach as used in the first transsphenoidal operation by Herman Schloffer 1907 with nose flected to the side.

The first transsphenoidal approach to the hypophysis in humans was elaborated in Innsbruck, Austria, by the surgeon Hermann Schloffer in 1906 [57]. He reported on the success of this operation in 1907 [58]. In his original work, he used a very traumatic and cosmetically unfavourable superior transsphenoidal approach with nose flected to the side and removing all endonasal bony structures (Figure 8). Modifications of this very invasive and mutilating method with the aim of reducing the operative trauma were performed by Anton von Eiselsberg (1860–1939), Head of the First Surgical Department at the University of Vienna in 1907. von Eiselsberg reported on his experience with this approach at the influential North American Surgical Society Meeting in 1910 [59]. He had the greatest surgical experience with this approach with 36 patients. His counterpart Julius Hochenegg (1859–1940), Head of the Second Surgical Department at the same University of Vienna, used this approach in 1909 to treat successfully the first case of acromegaly [60]. In the same year, Theodor Kocher (1841–1917) reported on his experience with the first operated case of a pituitary tumour using a submucous transseptal transsphenoidal approach preserving the middle turbinate and the ethmoidal cells [61]. At that time, Kocher was the head of the surgery in Bern, Switzerland, and obtained the Nobel Prize for his contributions to physiology and surgery of the thyroid gland in 1905. From our present point of view, all these superior transsphenoidal approaches were so destructive that—despite their principal extracranial route through the nose—they cannot be called minimally invasive.

The first transsphenoidal approach which deserves to be named minimally invasive was developed again at the University of Vienna—but this time by ENT surgeons. Forerunner of the endonasal transsphenoidal approach to the pituitary tumours was the Hungarian stemming Markus Hajek (1861–1941). With his publication “*Pathology and therapy of inflammatory diseases of the nose and paranasal sinuses*” in 1899, he belongs to the founders of rhinology. Hajek published an endonasal approach to treat abscesses in the sphenoidal sinus and the posterior ethmoidal cells in 1904 [62]. With the outbreak of the Second World War, he was forced to leave Austria due to his Jewish origin in 1939, and he died in exile in London in 1941. For Oskar Hirsch (1877–1965) working in Vienna at the same department as Hajek, it was then only a small step to extend Hajek's approach through the sphenoidal sinus also into the sella turcica. Hirsch presented in an anatomical study this approach for treatment of pituitary tumours during a session of the Viennese medical society 1909 [63]. Later he modified the approach and used an endonasal submucosal rhino-septal route. He performed the first operation 1910 under local anesthesia in several sessions. The first report with two successful operations was published by him in 1910 [64] and the results of 12 operated patients in 1911 [65]. Later he performed the operations in one single step, and he completed the treatment in case of inaccessible tumour remnats by local radiation therapy. Hirsch fled from the Nazis to Boston in 1939 and worked there as a consultant for ENT surgery at the Massachusetts General Hospital. He comprised his surgical experience with pituitary tumours in his late publications with a review of 277 operations in 1952 [55] and of 413 operated pituitary tumours in 1956 [66].

Simultaneously to Austria in the first decade of the 20th century, there was also a great interest in pituitary surgery in the United States. Harvey Cushing (1869–1939) studied the physiology and the anatomy of the pituitary gland in the Hunterian Laboratory at John Hopkins University in Baltimore since 1904.

The first operation of a pituitary tumour in the States was performed by Allen Buckner Kanavel (1874–1938) in Chicago at the Northwest University introducing the inferior transsphenoidal approach to the pituitary through a skin incision under the nose in 1909 [67]. One of the operated patients was suffering from a pituitary tumour and the second from a malignant tumour at the skull base. Samuel Jason Mixter (1855–1926), working as a surgeon at the Harvard Medical School in Boston, operated by the same method as Kanavel on a man with pituitary tumour and clinical signs of infantilism in 1910 [68, 69]. Samuel Mixter became known also for the first posterior interbody fusion at C1/C2. Later he and his son William Jason Mixter (1880–1953) worked together as neurosurgeons at the Harvard Medical School. The son became later known for the first endoscopic ventriculostomy and the first description of disc herniation. The first who applied a cosmetically satisfactory sublabial oronasal transsphenoidal approach was William Stuart Halstead in 1910 [70]. Halstead (1852–1922) was one of the outstanding surgical personalities in the United States of America at the turn of the 19th century. He spent two years till 1880 in Europe at the beginning of his academic carrier. He was impressed there with the basic medical sciences and the medical education. He had contact there to the most known anatomists, pathologists, and surgeons of that time such as Emil Zuckerkandl, Theodor Billroth, Jan Mikulcz-Radecki, and Hans Chiari. Back in the States, he realized his concept of surgery, science, and education at the John Hopkins University in Baltimore where he was one of the four founders of the medical faculty in 1892. He was the first to establish an educational program for surgeons in the States. Outstanding neurosurgeons like Harvey Cushing (1869–1939) and Walter Dandy (1886–1946) absolved their residency and the surgical training in the John Hopkins hospital and worked there in the Hunterian laboratory—Cushing in the field of endocrinology from 1904 till 1912 and Dandy thereafter performing basic work on CSF circulation. The first operative results from the United States and from Europe were vividly discussed at the American Surgical Society Meeting in 1910, and this discussion of papers of von Eiselsberg, Halstead, Mixter, and Quackenboss was believed to be so important that it was published by Cushing and Kanavel in the same year [71]. Cushing performed by chance in the same month as Oskar Hirsch in Vienna the first sublabial transsphenoidal approach to the pituitary in 1910. Initially Cushing used a modified superior transsphenoidal approach, later the inferior transsphenoidal approach, and finally he gave up the transsphenoidal approach at all and turned to the subfrontal transcranial approach because of less complications less recurrences, and better visual control [72, 73]. At that time, the handling of CSF leakages was problematic and the lack of antibiotics worsened the outcome of patients operated on by a transsphenoidal route. The authority of Harvey Cushing brought about that the transsphenoidal approach to the pituitary tumours was more or less given up for many decades. Only Norman Dott (1897–1973) [74] in Edinburgh, a pupil of Cushing equipped with a light at the speculum tip, and Gerard Guiot (1912–1998) [75] in Paris, using fluoroscopy for better orientation and cisternal pneumography to visualize intraoperatively the suprasellar tumour contours, continued with the transsphenoidal approach to pituitary tumours.

The turning point toward a modern era of transsphenoidal hypophysis surgery started with the application of the microscope to this surgery by the Canadian Neurosurgeon Jules Hardy in 1965 [76, 77]. Microsurgery with improved light also in narrow spaces, such as nasal cavities, contributed essentially to a reintroduction and broader application of the transsphenoidal approach. By means of the microscope a separation of tumour from pituitary gland became possible [78], and Hardy proposed the concept of a microadenoma instead of diffuse hyperplasia of the pituitary gland. In 1968 Hardy enlarged the operative indications and performed a selective anterior hypophysectomy in the treatment of diabetic retinopathy [79]. He developed also suitable instruments in bayonet shape to work with despite coaxial light transmission. The last step consisted in the introduction of endoscopic technique for this procedure by Jho and Carrau in Philadelphia [80] and Cappabianca [81] in Naples which enabled even better illumination and superior control in the deep nasal cavity than with a microscope. Beyond that, the endoscopic technique made it possible to look around the corner in the cavernous sinus and behind the carotid artery with application of angled optics. Since the initial introduction, many different groups all over the world popularized the endonasal technique more and more. The further development of this technique is still ongoing. In this paper only an overview about the history of pituitary surgery is possible. For more detailed information on history of pituitary surgery, I refer the reader to the excellent historical papers on this topic by Liu et al. [82], Lanzino and Laws Jr. [83], and Landolt [54].

## 4. Endoscopic Treatment of Hydrocephalus 

**Figure 9:**
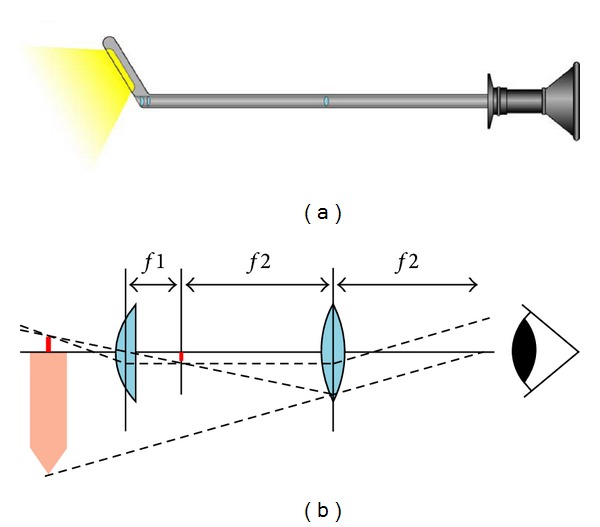
Nitze endoscope. (a) Nitze endoscope with a light source at the tip of the instrument and lenses at the tip and approximately at the middle of the shaft. (b) Principle of the Nitze endoscope. The optic is a Keppler telescope producing a virtual zoomed upside down image.

In the second half of the 19th century, Maximilian Nitze, a German physician, developed an endoscope which was serially produced and used as cystoscope in urology at the beginning of the 20th century [84–88]. Nitze's endoscopes had an optical system based on the principle of a Keppler telescope (Figure 9) which produced a virtual, zoomed, and upside down image. The light source was a platinum wire on the tip of the endoscope and required a special cooling system to prevent burning. Nitze developed the first prototype 1866 in Vienna together with the instrument maker Joseph Leiter in the department of surgery under the chairman von Dittel who was deeply intersted in the application of endoscopes in urology [89].

This endoscopic technology was brought across the Atlantic by the German instrument maker Wappler who founded the American Cystoscope Makers Incorporation (ACMI) in 1890. Thus, slightly improved Nitze-type endoscopes with an additional lens to reverse the upside down view were available at the beginning of the 20th century at the American universities. They were mainly applied in urology, gynaecology, and abdominal surgery. In 1910 at the Northwestern University in Chicago, the urologist and founder of andrology Victor Darwin L'Espinasse (1878–1946) and the neurosurgeon Allen Buckner Kanavel (1874–1938) performed the first endoscopic treatment of hydrocephalus by fulguration of the choroid plexus in the lateral ventricle [90]. However, they gave up this procedure after the second child died immediately after the the operation. Simultaneously in 1922 and 1923, Walter Edward Dandy (1886–1946) at John Hopkins University in Baltimore, Temple Fay (1895–1963) with Francis Clark Grant (1891–1967) at the Temple University in Philadelphia, and William Jason Mixter (1880–1958) in Massachusetts General Hospital in Boston made their first experiences with the endoscope in the treatment of hydrocephalus. Dandy called the endoscope due to its neurosurgical application a ventriculoscope [91]. He recognized the potential of this new technology for neurosurgery but because of technical limitations of the available endoscopes he preferred an open surgical approach to treat hydrocephalus [92–94]. Fay performed only an inspection of the ventricles in one single case and performed photographs of the ventricles. During the procedure he had however to switch to an open procedure. He published his experience with the endoscope and discussed the potentials and the limitations of this method [95]. Mixter performed the first endoscopic ventriculocisternostomy with the same method as it is performed today in 1923 [96]. All of these neurosurgeons gave the endoscopic procedure up due to technical limitations after few trials. Only John Edwin Scarff (1898–1978) who was resident in Baltimore with Dandy continued later at the Columbia University in New York with the neurosurgeon Byron Stookey (1887–1966) to treat hydrocephalus in case of obstructive hydrocephalus by ventriculocisternostomy and in case of malresorptive hydrocephalus by endoscopic fulguration of the choroid plexus [97–99]. The success rate of endoscopic fulguration of the plexus was about 50% and the mortality between 5%–15%. Thus, as soon as shunt system became available in the 50th, the implantation of these valves became the preferred method in the treatment of any type of hydrocephalus. Endoscopic techniques were given up for decades.

**Figure 10:**
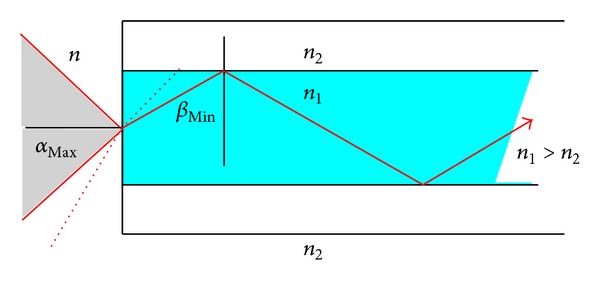
Flexible Hopkins fiberscope. The light is propagated in every fiber by total reflection due to the higher refraction of the glass fiber in relation to its cladding isolation.

**Figure 11:**
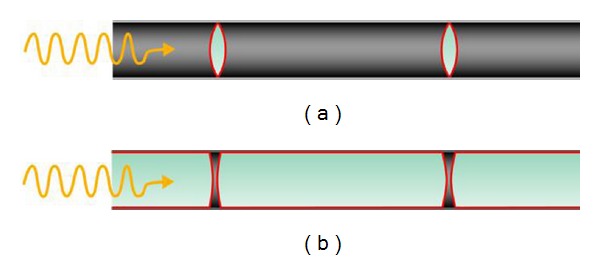
Principle of a rigid endoscope. (a) Standard rigid endoscope with high air to glass ratio. (b) Rod lens Hopkins endoscope. The reverse relation of glass to air and better coating of the lenses improves the light transmission 80-fold in comparison to the standard endoscope.

The key turning point in the application of endoscopy in neurosurgery was the development of optically improved endoscopes by Harold Horaz Hopkins (1918–1994). Hopkins was Professor of physics at the University of Reading, UK [100, 101]. At the beginning of the 50th, he met at a dinner party a physician complaining about the restricted flexibility of the endoscopes [102]. Hopkins had the idea to use bundles of glass fibres for light conduction by total reflection. This guaranteed a propagation of the light over a longer distance. For this purpose the glass fibres had to be cladded by a material with lower refraction than the glass. Hopkins published the prototype in 1954 in the journal Nature [103] (Figure 10). However his invention found no interest, and he had no money to develop the flexible endoscope to the final product. Hopkins' idea was picked up by the gastroenterologist Basil Hirschowitz from Ann Arbor, USA, who improved the flexible endoscope and presented it for the first time at a gastroenterological meeting in Colorado in 1957 [104]. Due to the negative experience from the previous invention, Hopkins hesitated to give his consent when he was asked to improve the image quality of rigid endoscopes in 1960. Finally, he agreed to this project, and he constructed 1961 a rigid endoscope with improved image quality, which allowed 80 times better light transmission than the standard endoscopes of that time. This could be achieved by replacing the standard lense with rod lenses which reversed the proportion of air to glass and thus increased nine fold the light transmission (Figure 11). A special cladding of the lenses contributed additionally to a better light conduction. The presentation of this endoscope during a urological meeting in Rio de Janeiro in 1961 was followed again by the same ignorance as his first invention. In 1964, Hopkins was invited to Düsseldorf, Germany, where he presented again the images of his rigid endoscope. This time Karl Storz, the Head and Founder of the Karl Storz Company, Tuttlingen, Germany, attended the meeting, and he realized immediately the great potential of Hopkins invention. A fruitful cooperation began. Storz contributed with an external “cold light” to the final product. The serial production of these endoscopes started in 1967.

In neurosurgery, the advanced endoscopes were used first for diagnostic purposes in the ventricles. Pioneers of the application of the advanced endoscopes in neurosurgery were Gerard Guiot (1912–1998) in Paris [105], Takanori Fukushima in Japan and later in Raleigh, US [106], and Hugh Griffith (1930–1993) in Bristol, UK [107]. In the 80th, endoscopic ventriculostomies were performed by few neurosurgeons scattered around the whole world—Nigel Jones and Charles Teo in Australia [108, 109], Kim Manwaring and Patric Kelly in the United States [110], and Christian Saint-Rose in Paris [111]. A strong impetus towards a common indication, technical development, with comparable experience and clinical results came at the beginning of the 90th from 3 centres in Germany: from Mainz around Axel Perneczky (1945–2009) [112–114], from Marburg by Dieter Hellwig and Berhard Bauer [115], and from Greifswald by Michael Robert Gaab, Henry Schröder, and Joachim Oertel [116] and from two Benelux countries by Andre Grotenhuis from Nijmegen [117] and by Jaque Caemaert from Ghent [118]. These centres successfully integrated endoscopy as a surgical tool in the concept of the minimally invasive neurosurgery. It is at present a recognized neurosurgical technique to treat obstructive hydrocephalus and further some lesions in or around the ventricles and in combination with microsurgery to check during the procedure the anatomy also around the corner.

## 5. Functional Stereotaxy 

**Figure 12:**
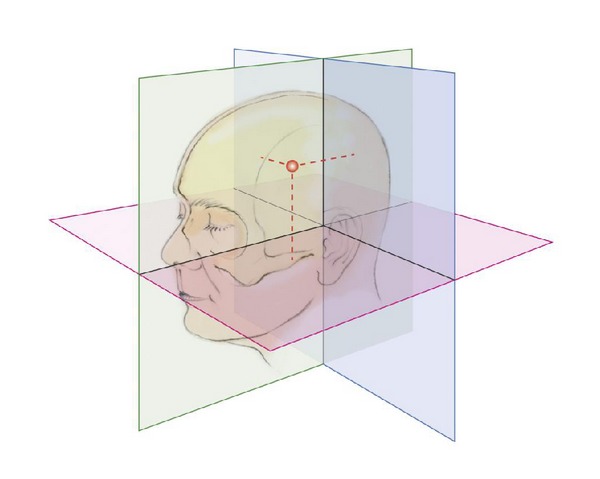
Clarke's concept of Cartesian localization. Every point is defined as a distance to three perpendicular planes. Horizontal plane defined as inferior orbital plane running through both acoustic meatus. The frontal plane is perpendicular to the horizontal plane and running through both acoustic meatus. Sagittal plane is perpendicular to the two other planes and divides the head in the midline in two equal parts.

Originally stereotaxy was developed by the physician Robert Henry Clarke (1850–1926) who worked together with the pioneer of British Neurosurgery Victor Horsley (1857–1916) at the University College in London since 1880. Both scientists were interested at the beginning of the 20th century in the contribution of the cerebellum and the deep cerebellar nuclei to movement. For this purpose, they intended to place electrodes into these deep cerebellar nuclei in apes for electrical stimulation and then to set a precise electrolytic lesion without collateral injury at the target to control the localization. Clarke and Horsley started their project in the Laboratory of Pathological Chemistry at University College in London in 1903. It turned out that a precise placement of the electrodes was difficult due to lacking anatomical landmarks, due to the curvature of the skull, and due to the variable depth which depended additionally on the various thicknesses of the bone and the integument beyond. Clarke developed a method which was independent of these disturbing factors. The idea was to create an intersection of three perpendicular planes and to measure from each plane the distance to the target point (Figure 12).

**Figure 13:**
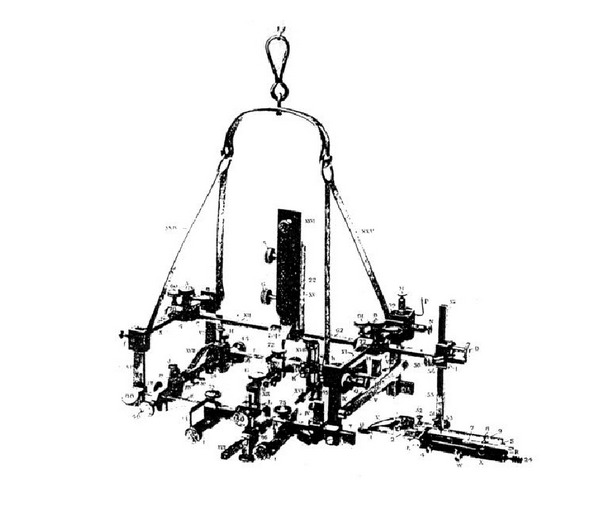
Clarke's stereotaxic apparatus for introduction of electrodes into deep nuclei for neurophysiological studies. The frame is fixed to the head and serves as a stable coordinate system and holder for the electrodes.

His long-winded formulation corresponds exactly to the concept of coordinates *x*, *y*, and *z* in a rectangular Cartesian coordinate system. Each distance from a plane coincides exactly with one of the three coordinates. Clarke was not a mathematician and he never used the term coordinates in his publication. In Clarke's formulation each point was in one of the 8 subspaces which were created by intersection of 3 perpendicular planes. A triple of 3 distances from each plane defined the position of the target. The planes had a constant way of construction. The first transversal plane was defined running from the low orbital line through each internal acoustic meatus. The second frontal or coronal plane was perpendicular to the transverse plane and was also running through each acoustic meatus. The third mid sagittal plane was perpendicular to the transverse and the frontal plane diving the head in the middle along the falx. The three distances to the anatomical structures could be read of from an atlas of the apes which were dissected exactly along the three perpendicular planes whose construction was described previously. To reach the target, a rectangular frame was constructed which was fixed to the skull prohibiting head movements during electrode introduction (Figure 13). The frame served also as a stable holder and carrier for the implanted electrodes. Electrolytic lesioning was performed with anodal current. The method was published first as a short report by Clarke as author in 1906 [119] and in detail as coauthor of Horsley in Brain in 1908 [120]. They called this method stereotaxis derived from the ancient Greek words stereos and taxis. Stereos means originally hard or solid but was also used as a technical term in the ancient mathematics to denote a special class of geometrical solids and also the third root of a number because in the ancient mathematics the third root could be visualized geometrically as a diagonal in a three-dimensional cube. Later stereos was used to denote spatial and three dimensional in general. Taxis means positioning or placement. Thus, stereotaxis should mean positioning in the 3-dimensional space. Clarke had also the idea to use this method in humans, but this did not occur before 1947. Clarke's idea was picked up by the Canadian anatomist and neurophysiologist Aubrey Mussen who worked with Clarke in London in the first decade of the 20th century. He bought also in London Clarke's stereotaxic system and used it for experimental studies on animals back in the Neurological Institute of McGill University in Montreal, Canada. Mussen's interest was in the anatomy of the brain stem and in particular the anatomy and physiology of the red nucleus and the cerebellum [121]. Mussen constructed also a stereotactic frame for human applications based on Clarke's principle [122]. However, this frame was never used. The stereotactic method had not gained a general acceptance for the application in humans before the Second World War. Although neurophysiologists such as Clarke or Mussen had the idea and also disposed of the necessary knowhow to perform stereotaxic operations in humans, but they did not have the corresponding surgical partners at that time to realize their ideas. The long standing surgical partner of Clarke, Victor Horsley, who would be able to perform a stereotaxic intervention, died in World War I in Mesopotamia in 1916. Mussen developed already a stereotaxic frame for human requirements but he could not convince a surgeon and a neurologist of the method to perform this operation with him. However, retrospectively it has to be admitted that due to the variability of the human skull in comparison to the animals the accuracy of Clarke's calculation method using only external bony reference structures would not have been sufficient for human applications. It is necessary to have additionally at least one internal reference point in the brain, and at that time, the imaging X-ray technique did not allow localizing intracerebral structures.

**Figure 14:**
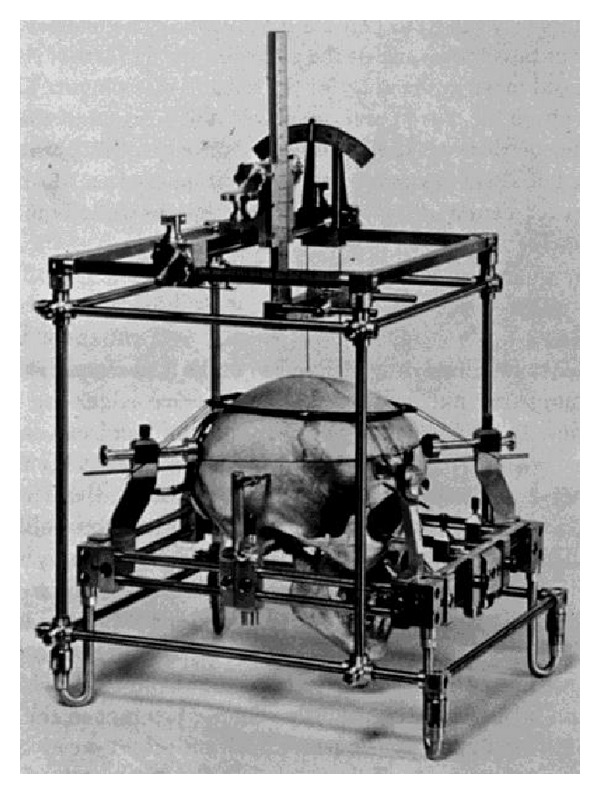
Stereotaxic apparatus of Spiegel and Wycis used during the first stereotactic procedures in humans.

Ernst Adolf Spiegel (1895–1985), who introduced the human stereotaxy, used an additional internal reference point—at the beginning just the calcified pineal body. Later, he used the posterior commissure as reference point because the pineal body was not always calcified and thus not always visible in radiographic images [123]. The visualization of the posterior commissure required a lateral image of the ventricles through ventriculography. This was achieved initially with air and later with diluted contrast media. Spiegel was originally born in Vienna in 1895. About his time in Austria is not much known. A publication on Hemitetanus in 1920 testifies his residency at the Neurological Institute in Vienna. In 1938, just before the occupation of Austria, he was a lecturer for physiology, neurology, and psychiatry at the University of Vienna. In 1939, he was forced to escape from the Nazis and fled to the United States where he worked at Temple University in Philadelphia as a neurologist. In his historical review on stereotaxy, Spiegel reported that he attended an open psychosurgical intervention in form of a lobotomy in 1947, and he was shocked with regard to the destructiveness of the procedure. He was convinced that this procedure with the same functional effect can be performed much less traumatically by a precise circumscribed lesion in the medial thalamic nucleus. In Philadelphia, Spiegel found in the neurosurgeon Henry Wycis (1911–1972) a skilful surgical partner who was able to realize this minimally invasive stereotaxic method for treatment of functional disorders [124–126]. It comprised the treatment of intractable pain, extrapyramidal movement disorders, epilepsy, and certain psychiatric disorders by means of accurate small circumscribed lesions of pathways or deep seated nuclei of thalamus, basal ganglia, or midbrain. These lesions were very effective and were much less invasive than open procedures in this region. At this time, the knowledge of anatomy, neurophysiology, and imaging technique with pneumoencephalography was so far advanced that an operation in these very eloquent areas seemed sufficiently accurate and safe. As the first stereotaxic operation, a lobotomy was performed with a modified Clarke's frame and the head as additionally fixed with a cast in 1947 (Figure 14) [123]. Only one year later, Spiegel and Wycis performed the first stereotactic operation on a patient with Parkinson's disease. Till the discovery of L-DOPA medication at the end of the 60th, the stereotaxic procedure constituted the only effective therapy of Parkinson disease.

Basically, the functional stereotaxic operations can be understood as procedures in the field of applied neurophysiology. Therefore, these procedures require a deep neurophysiological understanding of stimulation effects besides sophisticated mechanical equipment, good imaging technology, and knowledge of topographical anatomy. Therefore, stereotaxy started to flourish in neurosurgical departments where a close cooperation between a neurosurgeon, a neurologist, a neurophysiologist, a physicist, and an instrument maker was possible to establish.

**Figure 15:**
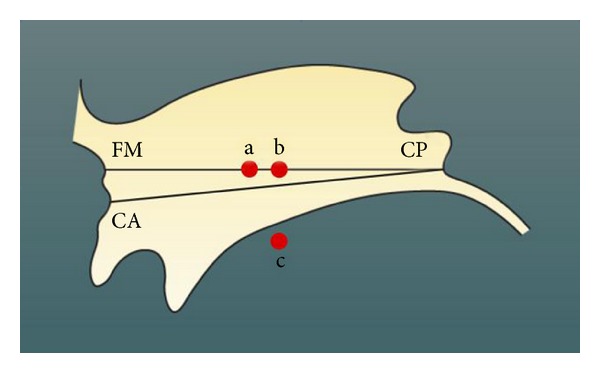
Third ventricle in lateral view with the stereotactic baselines for calculation: anterior-posterior commissure (CA-CP) line and the Foramen Monroi-posterior commisure (FM-CP) which was used in case the commissura anterior was not clearly visible. The red points mark the target points in relation to the baselines CA-CP and FM-CP. a: v.o.a. nucleus of thalamus for treating the rigor, b: v.o.p. nucleus of thalamus for treating tremor, c: Forel H field.

In Paris, it was the team around the neurosurgeon Jean Talairach (1911–2007) and the Neurophysiologist Jean Bancaud (1921–1993) who introduced stereotaxy in the L'Hôpital St. Anne in 1949 [127]. Talairach showed in his basic stereotactic research work in 1954 that Spiegel's calculations were only accurate for targets close to the reference point of the posterior commissure and became increasingly inaccurate the more the target was anterior to this reference point. Talairach introduced therefore in addition to the posterior commissure the anterior commissure. The distance connecting these two reference points—the CACP distance—became the stereotactic baseline for localization of deep brain structures independent of individual size and shape (Figure 15) [128]. Also in Paris, Gerard Guiot (1912–1998) established at the L'Hôpital Foch a stereotactic unit in 1958 where he worked together with Madame Denise Albe-Fessard (1916–2003) who was one of the most recognized specialists in neurophysiology of the thalamus and the basal ganglia. They introduced successfully 1961 during stereotaxic operations microelectrode recordings in the thalamus and were able to record tremor sensitive cells intraoperatively in Parkinson patients and thus to delineate the best target for treatment of tremor in the ventrointermediate nucleus (VIM) of the thalamus in [129]. Lars Leksell (1907–1986) at Karolinska Institute working under Herbert Olivecrona developed a target centred stereotactic frame in 1949. It enabled by positioning the target into the isocenter of the frame the surgeon to reach the target from any angle leading to a higher degree of flexibility for the trajectory [130]. In cooperation with Uppsala University cyclotron, he developed with the physicist Borje Larson the first stereotactic irradiation apparatus in 1951 [131]. He called this device due to its precision in Swedish language a “stralkniven” (ray knife). It was a prototype of the later gamma knife, which was developed in 1968 and is a sphere with fixed radiation sources whose gamma rays from all directions cross in the geometrical isocenter. By placing the target area into the isocenter, high dose local radiation can be applied which spares the surrounding brain tissue because the radiation is distributed through about 200 single radiation sources.

In Germany, the centre for stereotactic surgery became Freiburg starting in the 50th of the last century. Trautgott Riechert (1905–1983), the head of the neurosurgery, developed with the physicist and instrument maker Wolff a prototype in 1951 [132] and an advanced version of a very precise stereotactic system based on a polar coordinate system in 1953. The latter was additionally equipped with a phantom to check the coordinates and the trajectory [133]. This system was, due to its mechanical accuracy and the manually subtle instruments and electrodes, the most often used stereotaxic system worldwide till the 80th. The success of the Freiburg group would not be possible without the contribution of Rolf Hassler (1914–1984). He was a neuroanatomist and a specialist in the anatomy of the thalamus. Based on his anatomical knowledge a precise subdivision of thalamic nuclei was elaborated. Due to his work, the thalamus instead, of the basal ganglia, became more and more important for functional stereotaxy—particularly in the treatment of Morbus Parkinson [134]. Later he switched to basic research and was heading a laboratory for the study of Morbus Parkinson at the Max Plank Institute in Frankfurt.

Across the pacific, Spiegel's technique was picked up by Hirotaro Narabayashi (1922–2001) in Japan who developed with Uchimura a stereotactic frame based on the drawings of the stereotactic apparatus of Horsley and Clarke in Tokyo in 1949 [135]. The first successful stereotactic pallidotomy was performed by him in 1951. At this time, he was an Associate Professor of psychiatry at the Jutendo University in Tokyo. Later, he became Chairman of the Department of Neurology at the University of Tokyo. In 1957, he established a private clinic for stereotactic surgery which became a well-known research centre for movement disorders.

**Figure 16:**
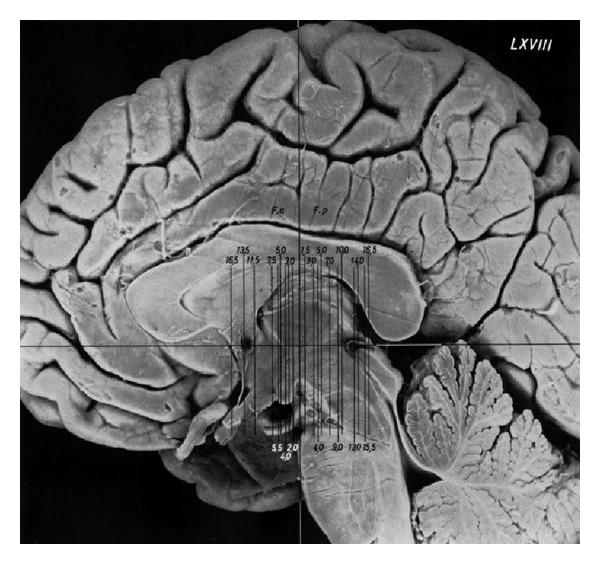
Schaltenbrand-Bailey Atlas. Lateral view with scout view of coronal sections of thalamus perpendicular to the stereotactic baseline (anterior-posterior commissure).

At this time, the topographical anatomy for stereotaxic procedures was supported by atlases of the deep brain structures. First atlas for stereotaxic applications was published by Spiegel and Wycis in 1952 [125] and 1962 [126]. Most known and used by the stereotaxic neurosurgeons became the Schaltenbrand-Bailey atlas published by the German neurologist from Würzburg Georg Schaltenbrand (1897–1979) and the pioneer of neuropathology and neurosurgery from Chicago Percival Bailey (1892–1973) in 1959 [136, 137]. A revised and enlarged version of this atlas was published together with Waldemar Wahren in 1977. The atlas was based on a cadaver study and consisted of 1–1.5 mm thin sections through the thalamus and the basal ganglia aligned to the CACP line in horizontal, sagittal, and frontal sections. The nomenclature of the detailed thalamic nuclei was influenced by the work of the German neuroscientist Rolf Hassler (1914–1984). The atlas was used by generation of stereotactic neurosurgeons to determine the coordinates of the desired targets in relation to the CACP line (Figure 16).

After the introduction of L-DOPA medication in the 70th the stereotactic surgery lost its most important clientele and the functional stereotactic operations were mostly given up except in few centres. However, 10 years later adverse effects of the drug therapy emerged such as on-off phenomena. Other extrapyramidal diseases such as essential tremor was also only partly treatable by drug medication. Therefore, since the 80th the stereotaxic treatment became again more and more popular. Instead of performing lesions, nondestructive treatment with chronic electrodes for stimulation was successfully introduced into the treatment of movement disorders using the same targets as for lesional stereotaxy. This deep brain stimulation (DBS) was in the 70th and mid 80th primarily used for the treatment of intractable pain. Alim Louis Benabid in Grenoble introduced DBS in 1987 in the VIM nucleus of thalamus for treatment of Morbus Parkinson [138], and Jean Siegfried in Zürich applied this technique in 1994 bilaterally in the pallidum [139]. Due to it effectiveness on tremor rigor and akinesia the Nucleus subthalamicus STN became, due to the work of Benabid in 1994, the preferred target for this disease [140].

## 6. Towards a Common Philosophy of Minimally Invasive Neurosurgery MIN 

The advanced endoscopic technology was primarily applied for diagnostic purposes by gastroenterologists, urologist, orthopaedist, and abdominal surgeons. 1976 these disciplines organized themselves in the US as “surgical study group for endoscopy and ultrasound.”

During the 80th, endoscopic procedures were increasingly applied in the abdominal surgery. The indication comprised inguinal herniations, appendectomies, cholecystectomies, and also tumors of the digestive tract. This new endoscopic surgery required the development of new surgical instruments and required an operative technique which was very different from the standard open operations regarding the visual control using only a screen and have an image distortion with a fisheye effect. The different handling of the instruments required a special training. This caused the endoscopic surgery to become increasingly recognized as an independent discipline. The British surgeon John EA Wickham coined for these procedures in 1984 the term minimally invasive surgery. He published his philosophy of this type of surgery in the British Medical Bulletin 1986 [141]. He understood the endoscopic surgery as a new minimally invasive method in opposition to the standard open operative procedures. In neurosurgery, the term minimally invasive neurosurgery acquired a slightly different interpretation. In neurosurgery as I showed in this paper in four examples from different neurosurgical fields, endoscopy was not the single technical development which contributed to making of a surgical procedure less traumatic and invasive. In neurosurgery converged with endoscopy, the neuronavigation, the stereotaxy, the 3D planning computer workstations, and the planning of individual microsurgical approaches based on the anatomical peculiarities and details of the pathology in all fields of neurosurgery toward a common philosophy of minimally invasive neurosurgery. The endoscope became only a useful tool during a part of the microsurgical procedure.

The neurosurgeon who deserves the greatest merits in developing and popularizing the minimally invasive technique for neurosurgery was at the end of the 80th Axel Perneczky (1945–2009), a Hungarian stemming consultant of neurosurgery at the University of Vienna. In Vienna he was at that time a recognized neurosurgeon in the field of vascular microsurgery. The philosophy of minimally invasive surgery suited very much his general neurosurgical concept. His ambition was to minimize as much as possible the intraoperative trauma for the patient respecting also the cosmetic aspects. This could be achieved by careful and thorough preoperative planning of the best approach which was based on a detailed analysis of individual anatomy and topographic relationships of the lesion visible in the radiological images. The lowest functional intraoperative trauma required sometimes new and unusual approaches such as the contralateral approach to the suprasellar region or through the lateral ventricle. The new approaches required detailed knowledge of topographic anatomy, which he acquired while working as demonstrator at the anatomical institute in Vienna and in the late 70th while working at the laboratory of Gazi Yasargil in Zurich. Perneczky understood neurosurgery as applied neuroanatomy. In 1988, Perneczky became Chairman of the Neurosurgery at the Johannes Gutenberg University in Mainz. There, he organized a team of neurosurgeons which started to realize his idea of minimally invasive neurosurgery. The clinical application preceded work in basic research. With his friend Manfred Tschabitscher, anatomist at the University of Vienna, Perneczky studied the anatomy of the ventricles and the basal cisterns from an endoscopic view [142]. The neurosurgeon Klaus Resch completed in Mainz the anatomical study by postmortem endoscopic inspections. He convinced also Perneczky of the superiority of rigid endoscopes over flexible endoscopes for neurosurgery due to the much better image quality. Additionally, a laboratory was established for anatomical studies on cadavers. The first meeting in Mainz in 1989 on the subject of minimally invasive neurosurgery was too early and not successful. The breakthrough of this new technique in neurosurgery was the international meeting in Wiesbaden in 1993 for minimally invasive neurosurgery. This meeting, organized by Perneczky, gathered for the first time all neurosurgeons involved worldwide in neuroendoscopy and was also attended by many internationally recognized neurosurgeons. Their basic approval of this new method had a stimulating effect for the irradiation of these new techniques and philosophy around the whole world. During this meeting a society for minimally invasive neurosurgery (MIN) was established with international conferences every 2 years and a journal with the same name. The spark of minimally invasive neurosurgery spread also to the industry and with Minop1 and Minop2 projects for development of minimally invasive technology, instruments became supported and sponsored by many companies. For Perneczky minimally invasive neurosurgery comprised not only endoscopy. The endoscope was only a tool during the operation or a part of the operation. Minimally invasive neurosurgery comprised all surgical instruments or devices and operative techniques which helped to diminish the intraoperative trauma including 3-D operation planning workstations, navigation devices, and endoscopes. He demonstrated that by the key hole effect also huge deep seated brain tumours can be satisfactorily controlled and removed through a small craniotomy [143, 144].

As I could show in this paper Perneczky was not the founder of minimally invasive methods in neurosurgery. These ideas started to be realized even by the pioneers of the neurosurgery. Interestingly, the great historic neurosurgical personalities were usually creative spirits not only in one but also in several neurosurgical fields. However, Perneczky deserves the merit of bundling and further developing all the new technical developments existent in the late 80th under a common neurosurgical concept of minimally invasive neurosurgery. Although Perneczky was one of the protagonists of this new philosophy, he would not be successful if not other neurosurgical centres in Europe and in other continents had at the same time simultaneously similar ideas and intentions. In particular in Germany it was the neurosurgery in Greifswald with Michael Gaab, Henry Schöder, and Joachim Oertel, in Marburg with Bernhard Bauer and Dieter Hellwig, and in the Benelux countries by Andre Grotenhuis, a close friend of Perneczky in Nijmegen and Jaque Caemmaert in Ghent who also essentially contributed to the development and promotion of this new surgical philosophy and technology in neurosurgery. All these groups exchanged their experiences during frequent international endoscopic, navigation, and keyhole surgery courses which were performed with the intention of education and hands-on training for neurosurgeons interested in this new field.
